# Chronic biopsy proven post‐COVID myoendocarditis with SARS‐Cov‐2 persistence and high level of antiheart antibodies

**DOI:** 10.1002/clc.23886

**Published:** 2022-07-19

**Authors:** Olga Blagova, Yuliya Lutokhina, Evgenia Kogan, Anna Kukleva, Dilara Ainetdinova, Vladimir Novosadov, Ruslan Rud', Polina Savina, Alexander Zaitsev, Viktor Fomin

**Affiliations:** ^1^ Department of Faculty Therapy No.1 N.V. Sklifosovsky Institute of Clinical Medicine, I.M. Sechenov First Moscow State Medical University (Sechenov University) Moscow Russia; ^2^ Department of Pathology N.V. Sklifosovsky Institute of Clinical Medicine, I.M. Sechenov First Moscow State Medical University (Sechenov University) Moscow Russia; ^3^ Department of Cardiology N2 of V.N. Vinogradov Faculty Therapeutic Clinic I.M. Sechenov First Moscow State Medical University (Sechenov University) Moscow Russia; ^4^ Department of Endovascular Methods of Diagnostics and Treatment I.M. Sechenov First Moscow State Medical University (Sechenov University) Moscow Russia

**Keywords:** antiheart antibodies, COVID‐19, endomyocardial biopsy, post‐COVID endocarditis, post‐COVID myocarditis, SARS‐Cov‐2, viral persistence

## Abstract

**Purpose:**

To study the clinical signs and mechanisms (viral and autoimmune) of myoendocarditis in the long‐term period after COronaVIrus Disease 2019 (COVID‐19).

**Methods:**

Fourteen patients (nine male, 50.1 ± 10.2 y.o.) with biopsy proven post‐COVID myocarditis were observed. The diagnosis of COVID‐19 was confirmed by IgG seroconversion. The average time of admission after COVID‐19 was 5.5 [2; 10] months. An endomyocardial biopsy (EMB) of the right ventricle was obtained. The biopsy analysis included polymerase chain reaction diagnosis of viral infection, morphological, immunohistochemical (IHC) examination with antibodies to CD3, CD45, CD68, CD20, SARS‐Cov‐2 spike, and nucleocapsid antigens. Coronary atherosclerosis was ruled out in all patients over 40 years.

**Results:**

The new cardiac symptoms (congestive heart failure 3–4 New York Heart Association class with severe right ventricular involvement, various rhythm, and conduction disturbances) appeared 1–5 months following COVID‐19. Magnetic resonance imaging showed disseminated or focal subepicardial and intramyocardial late gadolinium enhancement, hyperemia, edema, and increased myocardial native T1 relaxation time. Antiheart antibodies levels were increased 3–4 times in 92.9% of patients. The mean left ventricular (LV) ejection fraction (EF) was 28% (24.5; 37.8). Active lymphocytic myocarditis was diagnosed in 12 patients, eosinophilic myocarditis in two patients. SARS‐Cov‐2 RNA was detected in 12 cases (85.7%), in association with parvovirus B19 DNA—in one. Three patients had also endocarditis (infective and nonbacterial, with parietal thrombosis). As a result of steroid and chronic heart failure therapy, the EF increased to 47% (37.5; 52.5).

**Conclusions:**

COVID‐19 can lead to long‐term severe post‐COVID myoendocarditis, that is characterized by prolonged persistence of coronavirus in cardiomyocytes, endothelium, and macrophages (up to 18 months) in combination with high immune activity. Corticosteroids and anticoagulants should be considered as a treatment option of post‐COVID myoendocarditis.

## 1 INTRODUCTION

The COronaVIrus Disease 2019 (COVID‐19) pandemic has been going on for almost 2 years and largely influences routine clinical practice. The possibility of acute coronavirus‐induced myocarditis was demonstrated in a series of studies. The first detection of coronavirus particles by electron microscopy in myocardial biopsy of a patient with COVID‐19 and cardiogenic shock has been described,^1^ as well as identification of RNA virus in myocardium of patients with myocarditis.^2^ We have published a description of pancarditis in autopsy findings in patients with COVID‐19^3^; a little later, SARS‐Cov‐2 RNA was detected in the myocardium of all patients. However, descriptions refer only to cases of acute coronavirus myocarditis.

The maximum time since infection, in which RNA‐positive myocarditis has been detected, is only 4 weeks.^4^ The terms “post‐COVID,” “long‐term,” and “chronic” are not yet used in relation to coronavirus myocarditis. However, the ability of SARS‐Cov‐2 to induce prolonged, sustained over time, general and specific symptoms is accepted. This has given rise to the term “post‐COVID syndrome.” Inflammatory myocardial injury could be a component of this syndrome, which requires specific investigation using an endomyocardial biopsy (EMB).

### 1.1 Purpose

To study the clinical signs and mechanisms (viral and autoimmune) of myoendocarditis in the long‐term period after acute COVID‐19.

## 2 METHODS

Fourteen patients (nine male, 50.1 ± 10.2 y.o., range: 35–66 y.o.) with morphologically verified post‐COVID myocarditis were included in the study.

*Inclusion criteria* were a history of serologically verified new coronavirus infection, appearance or marked progression of cardiac symptoms (rhythm abnormalities, chronic heart failure [CHF]) after COVID‐19, presence of Dallas morphological and immunohistochemical criteria for active myocarditis according to ESC guidelines 2013.

*Exclusion criteria* were previously verified by MRI and/or EMB myocarditis, immunosuppressive therapy, coronary artery stenoses over 50%, valvular heart diseases, hypertensive heart disease, diffuse connective tissue disease, systemic vasculitis, sarcoidosis.

*Peculiarities of acute COVID‐19*. The diagnosis of COVID‐19 was confirmed by the appearance of SARS‐Cov‐2 IgG. PCR examination of nasopharyngeal smears was performed in 10 patients and was positive in four patients. Nonsevere bilateral viral pneumonia was diagnosed by chest CT scan in five patients; in eight patients chest CT was not performed. There were no cases of severe respiratory failure or evidence of acute cardiac injury during the acute COVID‐19. Treatment varied, but steroid therapy was administered in one patient only.

### 2.1 Comorbidities

The mean body mass index was 28.3 ± 3.5 kg/m^2^. Seven patients had a history of arterial hypertension, which was well controlled. One patient had a history of smoking, one—atopic bronchial asthma, one—diabetes mellitus, and one—bicuspid aortic valve without significant valvular dysfunction. Ten patients had no symptoms of heart disease before the COVID‐19. Other four patients had cardiac symptoms of unclear genesis before coronavirus infection (premature ventricular beats [PVBs] and atrial fibrillation [AF]) in two patients, and moderate left ventricular (LV) dysfunction in two patients]. The myocarditis was not previously diagnosed.

After COVID‐19, all patients experienced prominent cardiac symptoms. The mean time to clinic admission after COVID‐19 was 5.5 [2; 10] months, ranging from 2 to 9 months; the time to onset of symptoms after acute coronavirus infection was 1–5 months. In two cases, symptoms of myocarditis appeared not after COVID‐19, but only after subsequent vaccination.

### 2.2 Methods

EMB of the right ventricle (RV) was obtained. The analysis of myocardial biopsy specimens included hematoxylin‐eosin and Van Gieson stains, immunohistochemical (IHC) examination with antibodies to CD3, CD45, CD68, CD20, SARS‐Cov‐2 spike, and nucleocapsid antigens. This study was supported by Russian Foundation for Basic Research grant no. 20‐315‐90021/20. The specialized cardiac pathologist evaluated the all EMBs.

The myocardium was examined by polymerase chain reaction (PCR) for DNA of parvovirus B19, herpes group viruses, adenovirus, and SARS‐Cov‐2 RNA. Total RNA was extracted from myocardial fragments using the RNeasy Mini Kit (Qiagen, Germany). A QuantiTect single‐step PCR kit (Qiagen) was used to identify SARS‐CoV‐2. Primers selection was based on the publicly available data on gene DNA and mRNA sequences in the NCBI database, Primer‐BLAST software was used. The evaluation of antiheart antibodies (AHA) titers by indirect immunofluorescence method, echocardiography (EchoCG), 24‐h ECG monitoring, coronary angiography, and cardiac MRI (*n* = 6) were also performed.

*Statistical analysis* was performed using IBM SPSS statistics v.22.

### 2.3 Ethical approval

The investigation is conform to the principles outlined in the Declaration of Helsinki. All patients signed an informed consent to participate in this study, which was approved by the local ethics committee of Sechenov University.

## 3 RESULTS

### 3.1 The general clinical characteristics of the patients are presented in Table 1

**Table 1:** Clinical characteristics of patients with post‐COVID myocarditis

Parameter	Mean value
CHF class, NYHA	3 [3.0; 3.125]
white blood cells, х10^9^	6.6 [4.8; 8.0]
neutrophils, х10^9^	3.3 [2.5; 4.7]
lymphocytes, х10^9^	1.8 [1.4; 2.6]
hemoglobin (g/l)	147 ± 22
CRP (mg/l)	2 [1.0; 4.1]
LV EDD (cm)	6.1 ± 0.7
LV EDV (ml)	165 [142; 190]
LV ESV (ml)	107 [95; 137]
LV EF (%)	28 [24.5; 37.8]
Left atrium (cm)	4.5 ± 0.8
Left atrium (ml)	79 [67; 117]
Right atrium (ml)	49 [39; 82]
Right ventricle (cm)	3.5 ± 0.5
PASP (mmHg)	31.0 [26.5; 42.0]
Mitral regurgitation, grade	1.0 [1; 1.625]
Tricuspid regurgitation, grade	1 [0.875; 1.0]

Abbreviations: CHF,  chronic heart failure; CRP,  C‐reactive protein; EDD, end‐diastolic diameter; EDV, end‐diastolic volume; EF, ejection fraction; ESV, end‐systolic volume; LV, left ventricle; NYHA ‐ New York Heart Association; PASP,  pulmonary artery systolic pressure.

**Figure 1:**
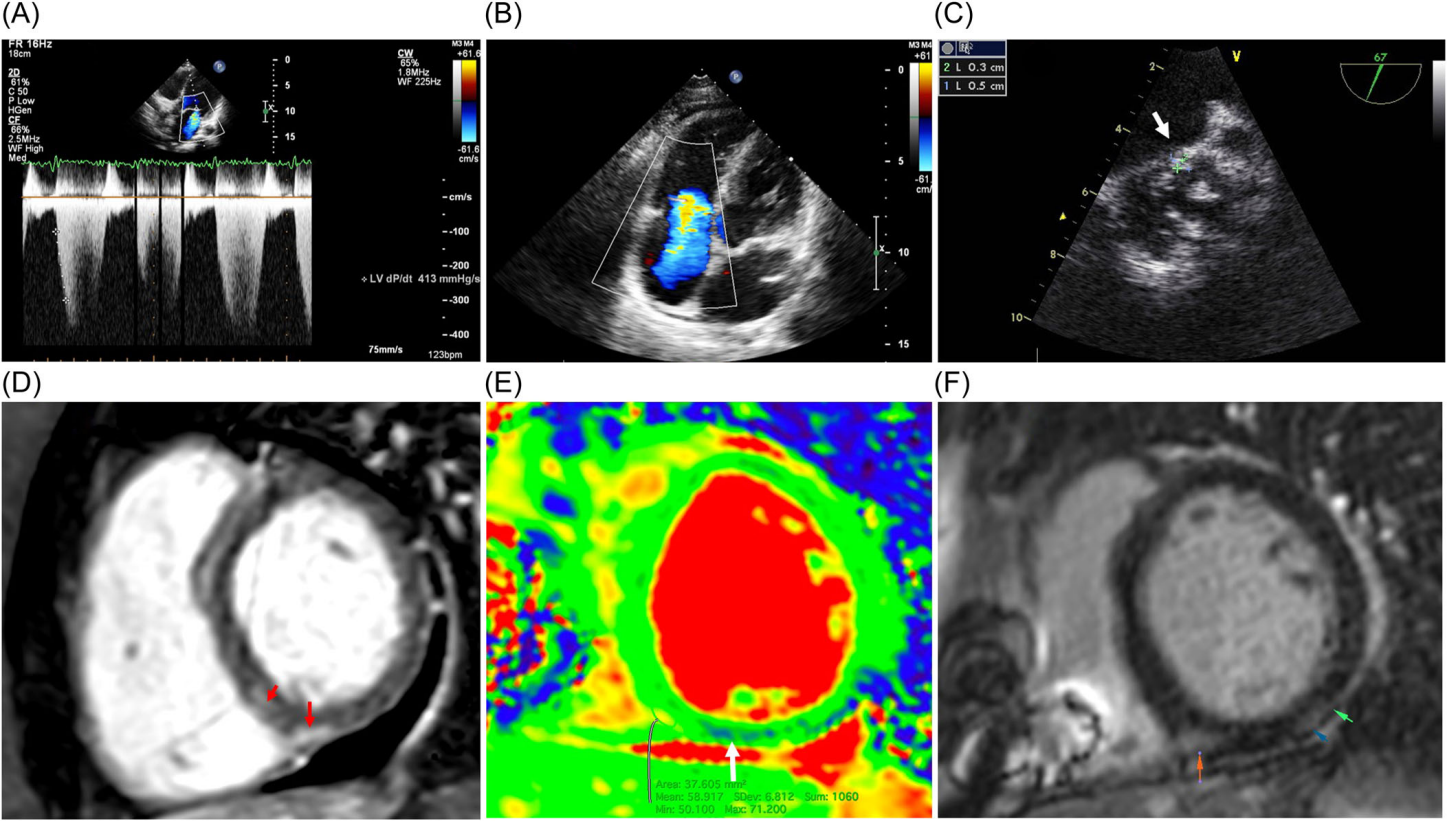
Echocardiography and magnetic resonance imaging in patients with post‐COVID myocarditis. Upper series—echocardiography: (A) decreased *dp*/*dt* (413 mmHg); (B) severe tricuspid regurgitation due to dilatation of the right ventricle; (C) vegetation on the bicuspid aortic valve measuring 3 × 5 mm (arrow), transesophageal study. Lower series—MRI: (D, F) late gadolinium enhancement in the posterior septal and posterior segments of the left ventricle (arrows); (E) edema along the posterior septal segment of the left ventricle (T2 map).

All patients were admitted for class 3–4 by New York Heart Association (NYHA) CHF. In 11 patients, it was biventricular (with peripheral edema, hepatomegaly, effusion in the cavities). We observed the moderate dilatation of all heart chambers and decreased LV ejection fraction (EF) up to 20%–30% (Figure 1A,B). High pulmonary hypertension and signs of pulmonary embolism were not found, which ruled out the secondary cause of myocardial dysfunction.

C‐reactive protein (CRP) elevation and leukocytosis remained in three patients. AHA titers were elevated 3–4 times (1:160‐1:320) in all except one patient. A typical ECG sign was a low QRS voltage (in 57.1%). Three patients developed persistent AF. Two‐thirds of the patients had PVCs and nonsustained ventricular tachycardia (VT). Two patients developed a left bundle branch block and another patient—AV block with pauses up to 5 s during AF.

On cardiac MRI only a patient with IE had signs of myocardial edema (Figure 1E), the others had 1–2 myocarditis criteria: subepicardial and intramyocardial late gadolinium enhancement mainly in LV myocardium and atria (Figure 1D,F), increased native myocardial relaxation time in T1 mode, increased extracellular volume, perfusion disorders and excessive amount of fluid in pericardium.

### 3.2 Results of morphological and IHC myocardial studies

A correlation of the clinical data of the patients with the myocardial morphological studies is presented in Table 2.

**Table 2:** Characteristics of patients with morphologically verified post‐COVID myocarditis

Parameters/patients	1	2	3	4	5	6	7	8	9	10	11	12	13	14
Gender	Male	Male	Female	Male	Male	Male	Male	Male	Female	Female	Male	Female	Female	Male
Age, years	56	64	45	44	39	45	66	43	62	47	35	47	65	47
CHF functional class (NYHA)	3	3	3–4	3–4	4	3	3	3	3	3	2	3	3	3
Time after COVID‐19 (months)	6	4	2	9	7	5	5	2	10	10	2	18	10	2
Postvaccinal symptoms onset	‐	‐	‐	‐	‐	‐	‐	‐	‐	+	‐	+	‐	‐
EMB results	LM	LM	LM	LM	LM	EM	LM	LM	LM	LM	LM	LM	EM	LM
Endocarditis by EMB	+	+	‐	‐	+	+	‐	‐	‐	‐	‐	‐	‐	‐
Thrombosis by EMB	Endocardium	Endocardium	Vessels	‐	‐	Endocardium	‐	‐	‐	Vessels	‐	‐	‐	‐
SARS‐Cov‐2 RNA in myocardium	‐	+	+	+	+	+	+	‐	+	+	+	+	+	+
Other viruses in myocardium	‐	‐	parvoB19	‐	‐	‐	‐	‐	‐	‐	‐	‐	‐	‐
CD3 lymphocytes per 1 mm^2^	15	15	10	12	40	12	10	13	18	10	7	16	10	14
CD45 lymphocytes per 1 mm^2^	20	20	15	35	60	25	35	18	20	17	32	24	17	32
Necrosis/cytolysis	+	+	+	+	++	++	+	+	+	++	+	+	+	+
Endotheliitis	++	‐	++	+	+	+	‐	+	‐	+	+	+	+	+
Fibrosis	+	+	+	+	‐	+	+	+	+	+	+	+	‐	+
Lipomatosis	‐	‐	‐	+++	‐	‐	++	+	+	‐	‐	++	++	‐
AHA level	+	+++	+++	++	+++	+++	++	++	++	+++	+++	++	++	++
Specific ANF	‐	‐	‐	1:80	1:160	1:80	1:80	1:40	1:80	‐	1:160	1:80	1:40	1:40
Low QRS voltage	+	+	++	+	‐	‐	+	‐	‐	+	‐	+	‐	‐
MRI (Lake Louise criteria)	na	+ (1)	Na	+ (2)	+ (2)	Na	+ (2)	Na	+ (1)	Na	Na	Na	+ (2)	‐
LV EDD (cm)	5.5	6.4	6.4	5.6	6.4	6.8	5.8	7.0	4.8	5.6	6.4	5.8	6.4	7.3
LV EDV (мл)	158	177	167	163	193	189	109	166	73	130	204	164	146	349
PASP (mmHg)	37	57	29	40	50	44	29	30	24	25	31	22	33	40
Initial LV EF (%)	43	27	21	20	25	27	26	19	40	25	33	37	29	22
LV EF dynamics (%)	46	48	47	41	42	45	49	38	58	47	38	57	43	55
Methylprednisolone (mg/day)	24	32	32	32	32	40	24	32	24	32	24	24	24	32
Implanted devices	‐	ICD	‐	‐	‐	‐	ICD	‐	‐	‐	‐	‐	‐	‐

Abbreviations: AHA, antiheart antibodies; ANF, antinuclear factor; CHF, chronic heart failure; EDD, end‐diastolic diameter; EDV, end‐diastolic volume; EF, ejection fraction; EM, eosinophilic myocarditis; EMB, endomyocardial biopsy; LM, lymphocytic myocarditis; LV, left ventricle; MRI, magnetic resonance imaging; parvoB19, parvovirus B19; Na, not available; NYHA, New York Heart Association; PASP, pulmonary artery systolic pressure; RNA‐ribonucleic acid.

**Figure 2:**
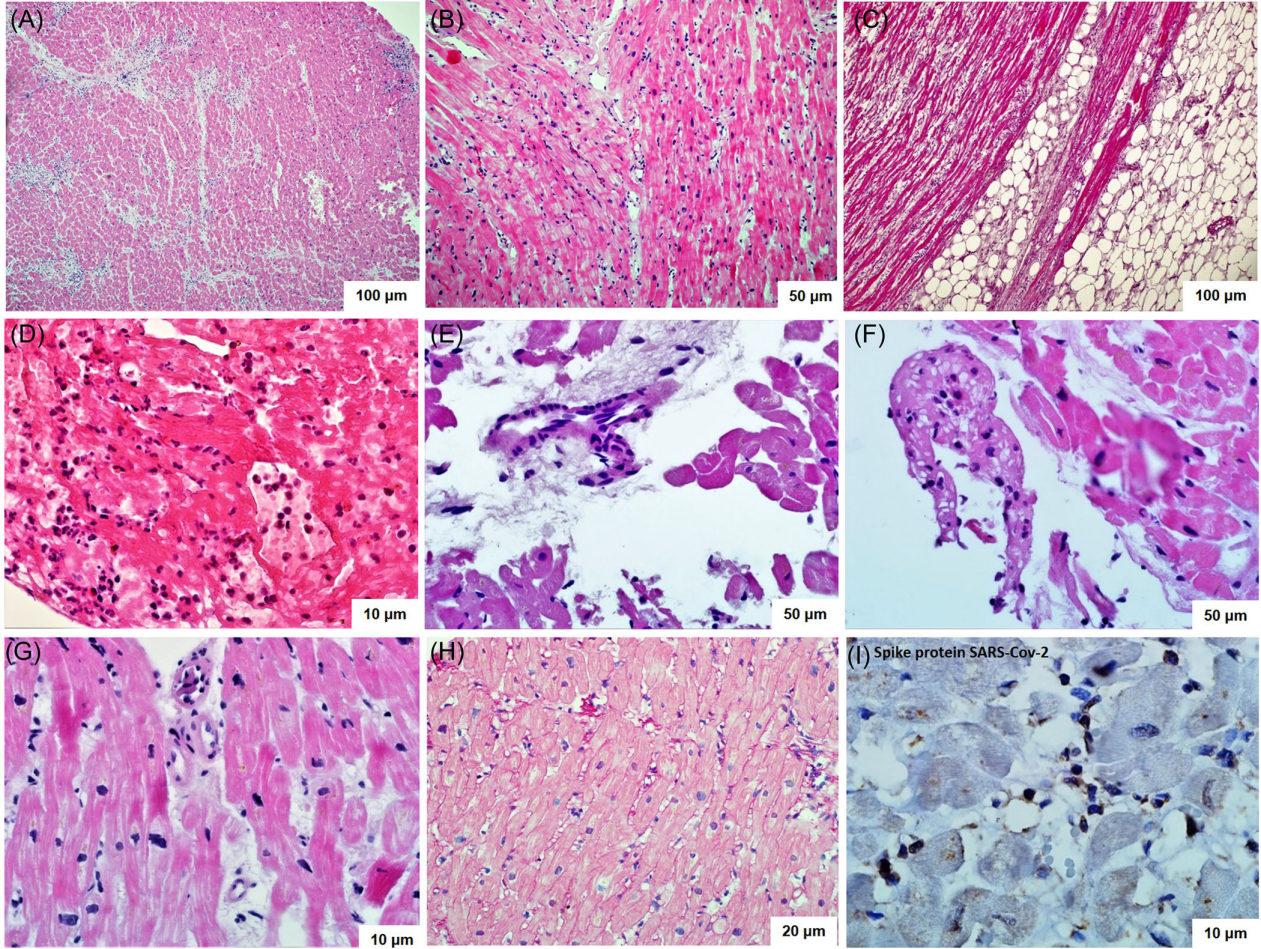
Endomyocardial biopsy of the right ventricle in patients with post‐COVID myocarditis. (A–G) Hematoxylin‐eosin staining: diffuse (A–D) and focal (G) infiltration with lymphocytes, giant multinucleated cells (B), eosinophils (D); cardiomyocyte necrosis with cytoplasm lysis, edema (A, B, D, G); areas of fatty replacement of the myocardium (C); endothelitis (E); microvascular thrombosis (G); fresh parietal thrombosis (F). (H) Van Gieson staining: diffuse perimuscular and perivascular sclerosis. (I) Immunohistochemical study with antibodies to the spike protein of SARS‐Cov‐2: positive reaction in vascular endothelium and infiltrate cells.

The diagnosis of active myocarditis was confirmed in all cases. The evidence of activity included signs of cardiomyocytes' death (necrosis, lysis) and severe dystrophy, as well as interstitial edema in all patients, Figure 2A,B,D,G. Cellular infiltrates were represented by lymphohistiocytic elements (Figure 2A–E,G) with eosinophis in one patient (Figure 2D). Coronariitis (endotheliitis) was detected in 79% of cases (Figure 2E). Two patients had microvascular thrombosis (Figure 2G) and three more patients had parietal thrombi in the RV (Figure 2F). The perimuscular and perivascular fibrosis was moderate (Figure 2H), which reflects the short history of myocarditis.

**Figure 3:**
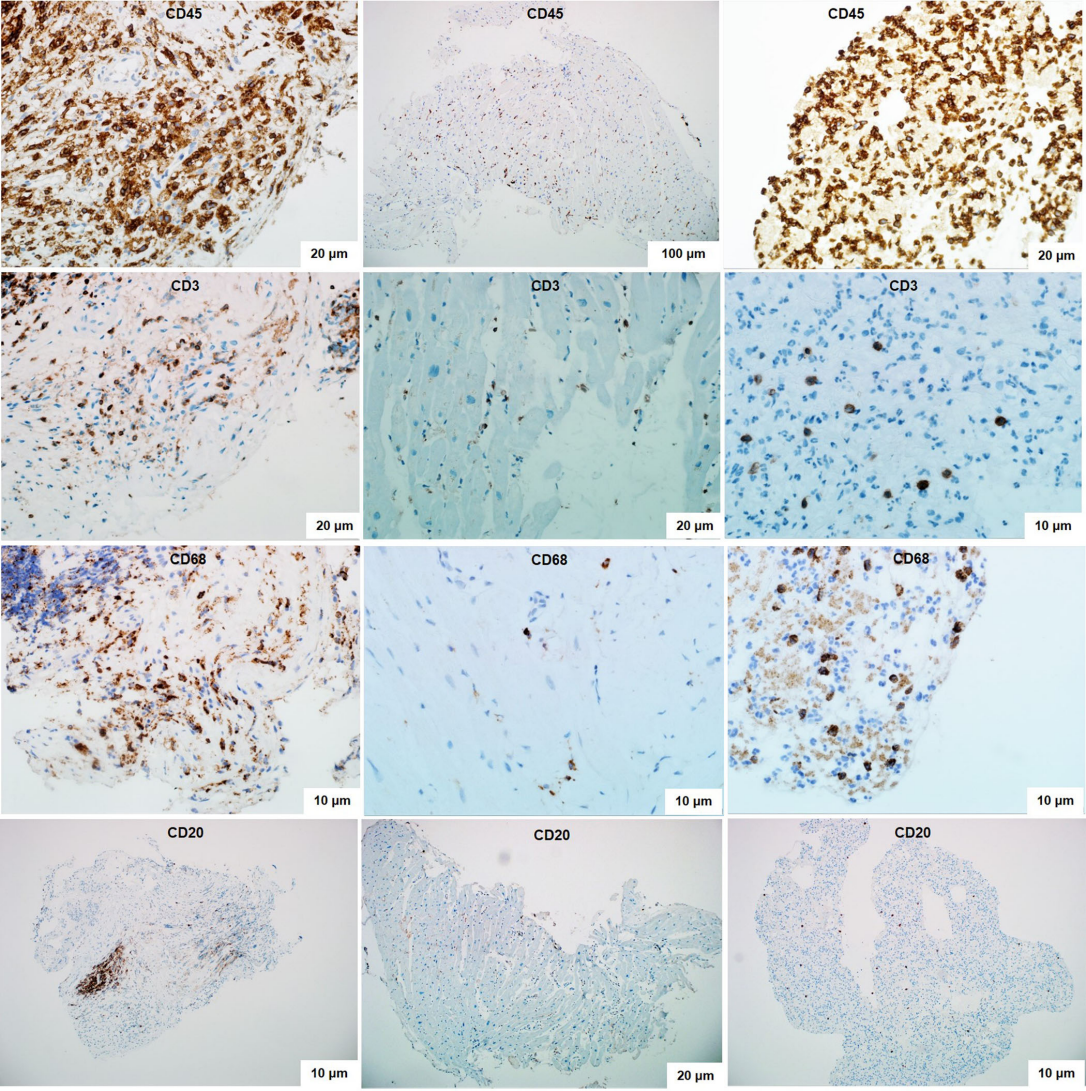
Immunohistochemical study of right ventricular myocardial biopsy specimens in patients with post‐COVID myocarditis. Diagnostically significant expression of CD45‐positive T‐lymphocytes (more than 14 cells per 1 mm^2^) and CD3‐positive T‐lymphocytes (more than 7 cells per 1 mm^2^). Various degrees of expression of CD68‐positive cells (macrophages) and CD20‐positive B‐lymphocytes.

IHC study confirmed the diagnosis of myocarditis in all cases (Figure 3). In some patients not only T‐lymphocytes, but also B‐lymphocytes and macrophages in varying numbers were detected in the infiltrates.

### 3.3 Results of virological (PCR and IHC) testing of myocardium

Real‐time PCR analysis of myocardium detected no adenovirus or herpetic virus genome. Parvovirus B19 DNA was found in one patient (see Table 2). SARS‐Cov‐2 RNA was detected in 12 from 14 patients. The maximal time after COVID‐19, when the virus was detected in the myocardium of a patient with active myocarditis, was 18 months (Table 2).

A pronounced positive expression to the SARS‐CoV‐2 nucleocapsid was detected in cardiomyocytes and infiltrate cells (mainly macrophages). Positive reaction to spike protein was observed in vascular endothelium and in infiltrate cells, including infiltrate in the endo‐ and pericardium, Figure 2I.

### 3.4 Endocarditis in patients with post‐COVID myocarditis

Concomitant endocarditis was diagnosed in four patients and manifested with two forms.

1. IE was diagnosed in a 39 y.o. patient with severe myocarditis (see Table 2) and a bicuspid aortic valve. The manifestations of IE included prolonged febrile fever, weight loss over 20 kg, progression of aortic stenosis and regurgitation, vegetations on the valve leaflets (Figure 1C), splenomegaly, severe increasing in inflammatory markers with negative blood culture. Progressive CHF with a persistent decrease of LV EF up to 25% was observed. EMB revealed an active lymphocytic myocarditis. Biopsy material culture and PCR analysis did not detect any bacteria.
2. Nonbacterial thromboendocarditis in three patients (see Table 2). There was no clinical suspicion of IE or parietal thrombosis (including MRI). Anticoagulant therapy was administered. EMB revealed signs of lymphocytic endocarditis with endocardial thickening and sclerosis, thrombotic masses. As well we observed a parietal thrombosis without endocarditis.

A common feature of patients with a combination of myo‐ and endocarditis was the detection of SARS‐Cov‐2 RNA in the myocardium and a low titer of antibodies to endothelium antigens in the blood, which may be considered as a consequence of escape of these antibodies to the endothelium as part of immune complexes. Other AHA titres were significantly elevated.

### 3.5 Treatment approaches for decompensated post‐COVID myocarditis

All patients received standard cardiotropic therapy at maximum tolerated doses, despite which severe myocardial dysfunction persisted. Anticoagulants were administered in eight patients (due to AF and/or thrombosis detected by biopsy). All patients were treated with corticosteroids, in one case of absence of coronavirus in the myocardium—in combination with mycophenolate mofetil 2 g/day. A good immediate response to treatment (increase in EF up to 47% [37.5; 52.5], improvement of CHF symptoms, decrease of inflammatory changes) was achieved (see Table 2). In five patients, the dynamics of EF has not yet been assessed due to a short period of observation (less than 3 months). Longer‐term follow‐up is required to assess remote outcomes.

## 4 DISCUSSION

This study presents a series of 14 cases of post‐COVID myocarditis, which manifested after the acute phase of COVID‐19, was diagnosed 2–18 months later and confirmed by myocardial biopsy. Only acute coronavirus‐associated myocarditis has been described so far. Further myocarditis progression is unknown. There are examples of rapid clinical improvement^5^ as well as fatal outcomes in virus‐positive patients.^6^ The issue of chronization of coronavirus‐associated myocarditis is the crucial one for long‐term prognosis.

There are almost no data on long‐term persistence of the virus in the myocardium and prolonged post‐COVID myocarditis. In the Charité‐Clinic a patient with CHF was described in whom SARS‐CoV‐2 genome and inflammation without necrosis were detected by EMB 4 weeks after the start of pulmonary symptoms.^4^ Three weeks later, a repeat EMB showed a reduction of inflammation and elimination of the virus. In another case, viral‐negative lymphocytic myocarditis was diagnosed by EMB one month after COVID‐19 and regressed rapidly.^7^ We are not yet aware of descriptions of more delayed SARS‐Cov‐2‐positive myocarditis.

Our study presents, for the first time, *long‐term (chronic) post‐COVID myocarditis*. SARS‐CoV‐2 RNA was detected in the myocardium of 12 out of 14 patients. The longest follow‐up after acute COVID‐19 was 18 months. Maximal late myocarditis was diagnosed in a patient who was vaccinated 1 year after acute COVID‐19. Only two such patients were included in the study. This suggests a trigger (resolving) role of the vaccine administered already after a coronavirus infection. At the same time, in both patients coronavirus was detected in the myocardium, which does not allow us to talk about isolated postvaccine myocarditis.

The absence of virus in one case demonstrates the virus‐negative post‐COVID myocarditis and other mechanisms for its development. This is primarily due to AНA production, which we detected in 73.5% of inpatients with coronavirus pneumonia in acute COVID‐19.^8^ In the present study, high AHA titers were detected in 93% of patients; they should be considered as pathogenetic mechanisms and markers of post‐COVID myocarditis.

To date, corticosteroids remain the only group of drugs for which a positive effect on prognosis in COVID‐19. Steroids resulted in a clear improvement in all of our patients. The feasibility of using anticoagulants in patients with myocarditis, myocardial microvascular thrombosis, and especially thromboendocarditis should be discussed.

## 5 CONCLUSION

SARS‐Cov‐2 infection leads not only to acute but also to subacute/chronic myocarditis, with clinical manifestations occurring within 1 to 4–6 months after acute COVID‐19. The main mechanisms of post‐COVID myocarditis are long‐term persistence of coronavirus in myocardium (the maximal period of SARS‐Cov‐2 detection after COVID‐19 was 9 months) combined with high immune activity (high titers of AHA). In a pandemic, any unclear myocardial dysfunction requires serodiagnosis of coronavirus infection. Corticosteroids and anticoagulants should be considered in the treatment of post‐COVID myoendocarditis.

## Data Availability

All data generated or analyzed during this study are included in this article.
